# Effect of Endometrioma cystectomy on cytokines of follicular fluid and IVF outcomes

**DOI:** 10.1186/s13048-019-0572-7

**Published:** 2019-10-21

**Authors:** Yu Liang, Xiaokui Yang, Yonglian Lan, Lingling Lei, Ying Li, Shuyu Wang

**Affiliations:** Department of Human Reproductive Medicine, Beijing Obstetrics and Gynecology Hospital, Capital Medical University, Beijing, 100026 China

**Keywords:** Endometriosis, IL-18, Ovarian endometrioma, Follicular fluid, Infertility, In vitro fertilization (IVF)

## Abstract

**Background:**

Endometriosis patients undergoing in vitro fertilization-embryo transfer (IVF-ET) treatment suffer from lower success rates. The success of IVF-ET is related to the receptivity of the uterus and the quality of embryos, and it is well known a patient’s endometriosis does not impair the receptivity. Whether endometrioma should be removed surgically before IVF remains controversial. Studies have shown that endometrioma removal decreases peritoneal inflammation, but little information is available regarding the alteration in the cytokines of follicular fluid. The objective of this study was to examine the impact of endometrioma cystectomy on the outcome of IVF and the levels of intrafollicular inflammatory cytokines and to investigate correlations between cytokine concentrations and IVF outcomes.

**Method:**

A total of 41 women with endometriosis-associated infertility undergoing IVF were recruited; 13 patients (surgery group, S group) had surgery to remove the endometrioma before enrollment, and 28 patients (non-surgery group, NS group) were untreated before IVF. The follicular fluid from a dominant follicle was collected during oocyte retrieval, and the concentrations of sixteen soluble cytokines known to be involved in ovarian function were measured.

**Results:**

Among the soluble molecules examined in this study, chemokines and growth factors and a few are inflammatory cytokines were found in the follicular fluid of patients with endometriosis. In addition, the expression levels of chemokines, growth factors, and most inflammatory cytokines did not differ between the S and NS groups, but interleukin (IL)-18 levels were significantly lower in the NS group. However, the levels of IL-18 in the FF did not correlate with IVF cycle parameters. The implantation and clinical pregnancy rates were similar between the two groups, but the anti-Müllerian hormone (AMH) level was lower in the S group than in the NS group.

**Conclusions:**

These findings suggest that endometrioma surgery may potentially reduce the ovarian reserve and has little impact on the success rate of IVF. Ovarian endometriomas are not associated with cytokine profiles in FF from infertile women, and they are not likely to affect the quality of the oocyte and embryo as a result of an inflammatory mechanism.

## Introduction

Endometriosis is a chronic gynecological disorder affecting more than 10% of women of reproductive age [1]. It is defined as the presence of endometrial glands and stroma outside the uterine cavity, causing chronic pelvic pain, dysmenorrheal, and infertility [2]. Endometriosis is strongly associated with infertility; several studies suggest that 20 to 50% of infertile women suffer from endometriosis and that 30 to 50% of women with endometriosis experience impaired fertility [3, 4]. Although several conditions have been associated with the occurrence of endometriosis, including anatomic distortion, decreased oocyte quality and impaired endometrial receptivity, the mechanisms of endometriosis-associated infertility are not fully understood [5].

It is widely accepted that endometriosis is a chronic inflammatory disease. Many studies have demonstrated that endometriomas lead to the recruitment of immune cells and an intense inflammatory response, with increased levels of proinflammatory cytokines, growth factors, and angiogenesis [6–8]. Jørgensen et al. (2017) measured a panel of 48 different cytokines from the peritoneal fluid of infertile patients and identified 13 cytokines that discriminate for the presence or absence of endometriosis [9]. These cytokines can be broadly considered as belonging to three subgroups with overlapping biological functions: chemokines (IL-8, MCP-1, MCP-3, and CTACK), hematopoietic growth factors (IL-5, IL-13, IL-9, M-CSF, and G-CSF), and general growth factors (HGF and LIF) [9]. Endometriosis is characterized by the increased number and activation of peritoneal macrophages and reduced cytotoxic effect of natural killer cells [10] and reactivity of T lymphocytes [11]. The increased number of activated peritoneal macrophages produce higher levels of pro-inflammatory cytokines, such as tumor necrosis factor-α, IL-6, and IL-1β. The reduced cytotoxic effect of NK cells can increase autoimmune reactivity, while the decreased T cell reactivity changes the Th1/Th2 balance toward Th2. Imbalanced Th1/Th2 may be related to endometriosis-associated infertility [12].

A damaging inflammatory milieu has also been proposed as a cause of diminished oocyte quality, which in turn could lead to poor IVF outcomes in patients with endometriosis [13, 14]. Follicular fluid [9] forms the biochemical micro-environment of the oocyte before ovulation and is used for estimating the developmental competence of female gametes [15]. The microenvironment of follicular fluid (FF) is closely associated with the formation of spindles and the distribution of chromosomes [16]. Singh et al. (2016) found a significant increase in the levels of pro-inflammatory (IL-1β, TNF-α, IL-2, IL-8, IL-12, and IFN-γ) and anti-inflammatory (IL-4, IL-6, and IL-10) cytokines in FF from women with endometriosis undergoing in vitro fertilization (IVF) as compared with controls [13]. Sarapik et al. (2012) found that IL-12 levels were positively correlated with oocyte fertilization and embryo development, while increased IL-18, IL-8, and MIP-1β levels were associated with successful IVF-induced pregnancy [17].

Endometriosis patients undergoing IVF treatment typically have a low success rate for establishing pregnancy [18–20]. The success of the IVF-ET process is related to the receptivity of the uterus and high-quality embryos, and it is well known that endometrial receptivity in eutopic endometrium in patients with endometriosis is not affected [21]. Evidence of the impact of an endometrioma on IVF is equivocal; the question of whether or not endometriomas should be surgically removed before IVF remains controversial [22, 23]. Previous studies have shown that endometriotic lesion removal decreases peritoneal inflammation [24], but little information is available regarding alterations of the intrafollicular inflammatory cytokine system. Because various cytokines in FF have been shown to play an important role in oocyte quality, including inflammatory factors, chemokines, and growth factors, it is important to investigate the impact of surgery on these cytokines.

The objective of this study was to examine the impact of endometrioma excision on the outcome of IVF and the levels of intrafollicular inflammatory cytokines in women with endometriosis, as well as to investigate correlations between cytokine concentrations and IVF outcomes.

## Materials and methods

### Patients

This prospective study was conducted between March 2017 and October 2018 at the Department of Human Reproductive Medicine, Beijing Obstetrics and Gynecology Hospital, Capital Medical University. Ethical approval was received from the local Institutional Ethics Committee and written informed consent was obtained from all participants. Women attending the center with indications for IVF treatment were recruited.

Forty-one patients with infertility due to stage III or IV endometriosis, which was diagnosed by ultrasound or laparoscopy, were recruited for this prospective, case-controlled study. The extent of endometriosis was staged according to the American Society for Reproductive Medicine classification of endometriosis. Among these patients, 13 (surgery group) were surgically treated for endometrioma before enrollment, and 28 (non-surgery group) were diagnosed with an ovarian endometrioma by ultrasound. All 13 surgical patients underwent a conventional laparoscopic cystectomy procedure.

The exclusion criteria were as follows: ≥40 years of age; body mass index (BMI) ≥30 kg/m^2^; basal follicle stimulating hormone (bFSH) concentration ≥ 12 mIU/mL; polycystic ovary syndrome; cycles with the dominant FF contaminated with blood during oocyte retrieval; cycles with the dominant FF not yielding oocytes; other endocrine diseases (thyroid disease, diabetes mellitus, and Cushing’s syndrome). The semen quality of the partners of recruited women was normal.

Within a month prior to starting the IVF stimulation, all patients underwent blood sampling to determine AMH levels using a standard enzyme-linked immunosorbent assay (ELISA; Beckman Coulter AMH Gen II, Brea, CA, USA; normal range = 1–8 ng/mL). In order to avoid the effect of surgery/analog treatment on AMH levels, the time between surgery and enrollment was more than three months.

### Controlled ovarian stimulation and IVF

Ovarian stimulation in all patients was initiated using the ultra-long gonadotropin-releasing hormone agonist (GnRHa) protocol, as previously described [25]. Pituitary desensitization was induced with the administration of 3.75 mg GnRHa (Triptorelin, Ferring, Germany) on day 2 or 3 of the menstrual cycle. After 28 days, patients underwent transvaginal ultrasonographic and biochemical evaluations. Once a suitable degree of downregulation was achieved (i.e., subjects had a serum oestradiol concentration ≤ 40 pg/mL, an endometrial thickness ≤ 5 mm, and arrested follicular development), human menopausal gonadotrophin (hMG; Menogon, Ferring GmbH, Kiel, Germany) or recombinant FSH (Puregon; Organon, Dublin, Ireland or Gonal F; Merck Serono S.p.A., Modugno, Italy) was administered with a starting dose of 150–300 IU per day based on the antral follicle count (AFC), age, and body mass index (BMI), according to standard operating procedures. Ovarian response was monitored by serial transvaginal scanning and hormonal monitoring. Gonadotrophin [12] dosage was further adjusted based on the ovarian response. When one to three leading follicles was ≥18 mm in diameter, 250 µg of human chorionic gonadotropin (hCG, Ovidrel, Merck Serono S.p.A.) was administered to trigger final maturation of the oocytes. Oocyte retrieval was performed 36 h later. Embryos were graded on day 3 according to a 1 to 3 consensus scoring system (with 1 being the top embryos), which was based on cell size and symmetry, fragmentation, multinucleation, and blastomere number (Alpha Scientists in Reproductive Medicine and ESHRE Special Interest Group of Embryology, 2011). Two embryos were transferred 3 days later. Excess good quality embryos were frozen for subsequent transfer. The luteal phase was supported with 90 mg of 8% progesterone gel (Crinone, Merck Serono) or 800 mg of micronized progesterone (Utrogestan, Laboratoires Besins International, Paris, France) daily. Progesterone support was initiated on the day of oocyte retrieval and continued for 14 days; the treatment continued for another 8 weeks if a pregnancy was achieved. A clinical pregnancy was identified 4–5 weeks after oocyte retrieval by the presence of an intrauterine gestational sac and a pulsating fetal heartbeat.

### FF collection and detection of cytokine profiles

FF was obtained from a single, large diameter dominant follicle during oocyte retrieval and stored at − 80 °C until further use. FF samples that were contaminated with blood were excluded. Sixteen selected cytokines/chemokines involved in inflammatory and angiogenic pathways in FF were detected by multiplex analysis using the Milliplex Magnetic Bead assay (Millipore, Billerica, MA, USA). The assay contained granulocyte colony–stimulating factor (G-CSF), granulocyte-macrophage colony-stimulating factor (GM-CSF), interferon (IFN) γ, chemokine (C-C motif) ligand 2 (CCL2), tumor necrosis factor (TNF) α, vascular endothelial growth factor (VEGF), CCL3, interleukin (IL) 1β, IL-2, IL-5, IL-6, IL-8, IL-10, IL-12(p70), IL-15, and IL-18. The Luminex 200TM system and Milliplex Analyst were used for detection and analysis.

### Statistics

Data were analyzed with the Statistical Program for Social Sciences (SPSS; version 18.0). Statistical comparisons were carried out using the Mann-Whitney U test, chi-square test, and Student’s t-test when appropriate. Pearson’s bivariate correlation coefficient analysis was performed to determine correlations. Pearson’s bivariate correlation analysis was performed to identify factors predicting IVF outcomes. A two-sided *P* < 0.05 was considered statistically significant.

## Results

### Clinical characteristics of patients and indexes of IVF treatment

There were no significant differences in age, duration of infertility, BMI, basal FSH levels or AFC between the surgery and non-surgery groups. The AMH level was lower in the surgery group (1.96 ± 1.00 ng/mL) than in the non-surgery group (3.71 ± 2.21 ng/mL, *P* = 0.01). There were fewer retrieved oocytes in the surgery group than in the non-surgery group (7.0 ± 5.69 vs. 12.32 ± 8.60, *P* = 0.05), but this difference was not statistically significant. The number, the size, and the bilaterality of the cysts did not significantly differ between the two groups (Table 1). Other IVF treatment characteristics (total Gn dose, medication used, E2 on HCG day, the number of MIIoocytes, fertilization rate, high quality embryo rate overall transferred, and endometrial thickness) and the implantation and clinical pregnancy rates were similar between the two groups (Table 2). These results suggest that endometrioma surgery may potentially reduce the ovarian reserve and has little impact on the success rate of IVF.

**Table 1:** Clinical characteristics for the patient groups

Parameter	Non-surgery (*n* = 28)	Surgery (*n* = 13)	P
Age (years)	31.57 ± 3.10	33.85 ± 5.47	0.10
Duration of infertility (years)	3.79 ± 2.54	4.23 ± 2.49	0.60
BMI (kg/m2)	22.72 ± 2.67	21.40 ± 2.47	0.14
Diameter of the cyst (cm)			
< 4 cm	9 (32.1%)	3 (23.1%)	0.72
≥ 4 cm	19 (67.9%)	10 (76.9%)	
Number of cysts			
single cyst	17 (60.7%)	7 (53.8%)	0.68
multiple cyst	11 (39.3%)	6 (46.2%)	
Endometrioma			
Unilateral	22 (78.6%)	9 (69.2%)	
Bilateral	6 (21.4%)	4 (31.0%)	0.70
bFSH (IU/L)	7.08 ± 1.74	7.97 ± 2.13	0.17
AMH (ng/ml)	3.71 ± 2.21	1.96 ± 1.00	0.01*
AFC	12.15 ± 5.51	8.69 ± 4.84	0.06

*BMI* body mass index, *bFSH* basal follicle-stimulating hormone, *AMH* anti-Müllerian hormone, *AFC* antral follicles count. Data are expressed as mean ± standard deviation. The *P* values were obtained from the Student’s t-test. The Chi-square test was used for the ratio analysis

*P < 0.05 was considered statistically significant

**Table 2:** IVF treatment characteristics for the patient groups

Parameter	Non-surgery (*n* = 28)	Surgery (*n* = 13)	P
Total Gn dose (U)	2012.03 ± 668.07	2243.46 ± 596.76	0.29
medication used			
rFSH	9 (32.1%)	5 (38.5%)	0.73
hMG	19 (67.9%)	8 (61.5%)	
E_2_ on HCG day (pg/ml)	3628.20 ± 2639.51	2399.7 ± 1082.21	0.13
Retrieved oocytes	12.32 ± 8.60	7.00 ± 5.69	0.05
MII oocytes	9.19 ± 6.98	6.08 ± 4.83	0.16
Fertilization rate (%)	(249/345) 72.2%	(74/91) 81.3%	0.08
High Quality embryo rate overall transferred (%)	91.1 (52/56)	91.5 (24/26)	1.0
Endometrial thickness [19]	10.71 ± 1.70	10.91 ± 1.85	0.65
Implantation rate (%)	32.6	26.9	0.79
Clinical pregnancy rate (%)	46.4	46.2	1.00

*Gn* gonadotropins, *E2* estradiol, *HCG* human chorionic gonadotropin. *HMG* human menopausal gonadotrophin, *MII* metaphase II. Data are expressed as mean ± standard deviation. The *P* values were obtained from the Student’s t-test. The Chi-square test was used for the ratio analysis

### Levels of cytokines in FF of women with endometrioma

**Fig. 1:**
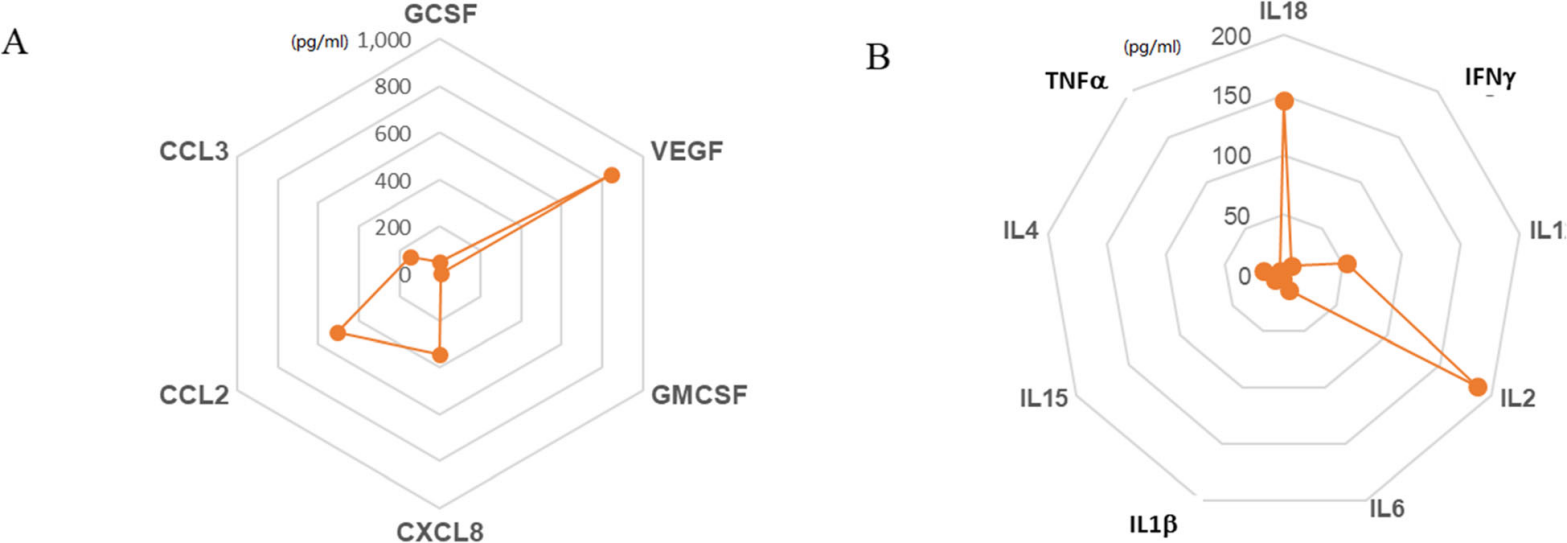
Cytokine profiles in FF of women with endometrioma. The radar charts show the median levels of G-CSF, GM-CSF, IFN-γ, IL-1β, IL-2, IL-6, IL-8, IL-12(p70), IL-15, IL-18, TNF-α, CCL2, CCL3, and VEGF in FF measured by a multiplex cytokine assay

The levels of cytokines, including G-CSF, GM-CSF, IFN-γ, IL-1β, IL-2, IL-5, IL-6, IL-8, IL-10, IL-12(p70), IL-15, IL-18, TNF-α, CCL2, CCL3, and VEGF, were determined in the FF of patients with endometrioma. The concentrations of IL-5 and IL-10 could not be quantified because they were below the detection level. The levels of the other cytokines are shown in radar charts (Fig. 1). Among the soluble molecules examined in this study, chemokines and growth factors (Fig. 1a) and a few are inflammatory cytokines (Fig. 1b) were the primary components found in the follicular fluid of patients with endometriosis.

### Impact of lesion removal on the levels of cytokines in follicular fluid

**Fig. 2:**
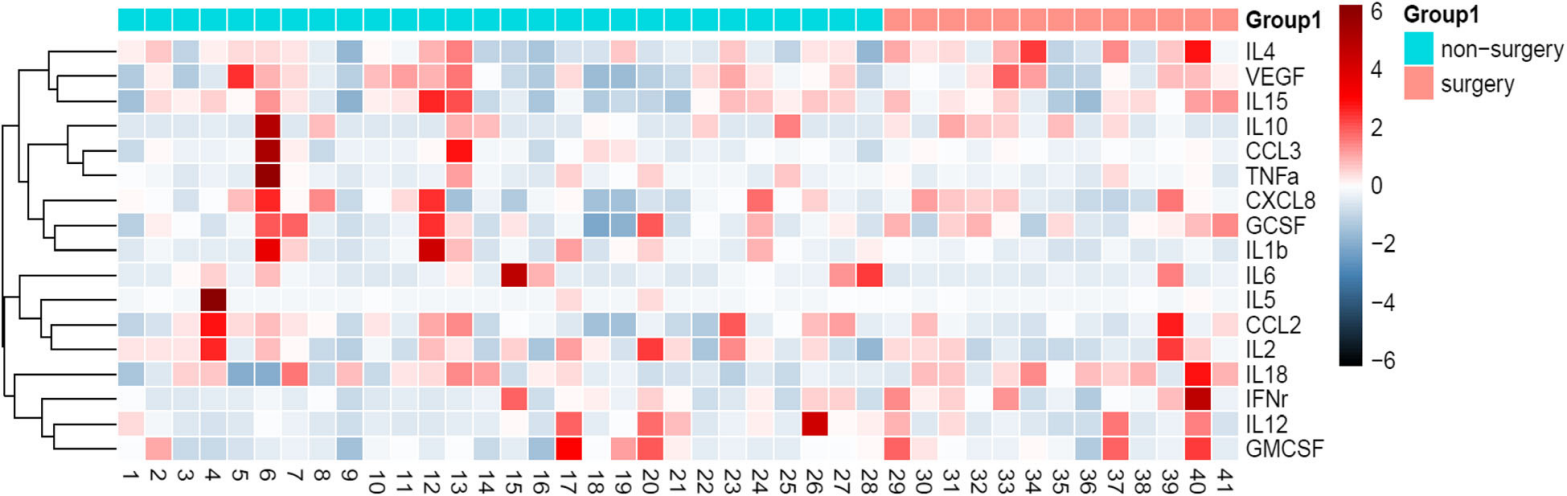
Heat map of cytokine levels in the patient groups. The levels of G-CSF, GM-CSF, IFN-γ, IL-1β, IL-2, IL-6, IL-8, IL-12(p70), IL-15, IL-18, TNF-α, CCL2, CCL3, and VEGF in FF were measured using a multiplex cytokine assay

**Fig. 3:**
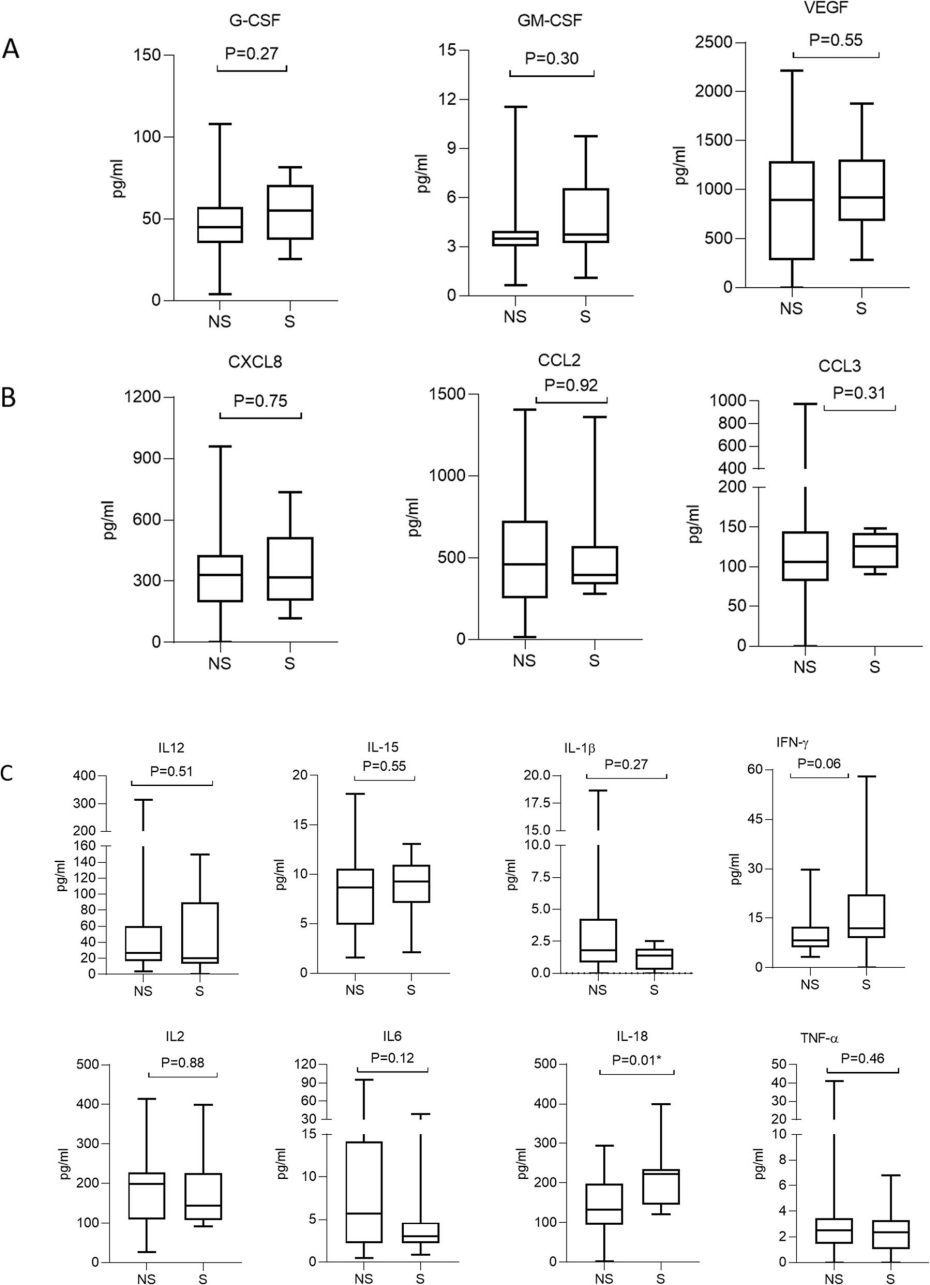
Concentrations of cytokines in the FF of patients. The cytokine levels in the FF from 13 patients in the surgery (S) group and 28 patients in the non-surgery (NS) group were analyzed. (**a**) Growth factors (G-CSF, GM-CSF, and VEGF); (**b**) Chemokines (CCL2, CCL3, and CXCL8); (**c**) Inflammatory factors (IFN-γ, IL-1β, IL-2, IL-6, IL-12(p70), IL-15, IL-18, and TNF-α). The horizontal lines in the box plots represent the median and the 25th and 75th percentiles. The *P* values were obtained from the Mann–Whitney U test

To explore whether or not lesion removal influenced the cytokine profiles in FF, we determined the cytokine levels in FF obtained from patients in the surgery (S, *n* = 13) and non-surgery (NS, *n* = 28) groups. The concentrations of the 16 cytokines in FF obtained from the two different groups are presented in Fig. 2. We also assessed differences in cytokine levels between the two groups. As shown in Fig. 3, the levels of chemokines (CCL2, CCL3, and CXCL8) and growth factors (G-CSF, GM-CSF, and VEGF) did not significantly differ between the two groups. In addition, the levels of inflammatory cytokines (IFN-γ, IL-1β, IL-2, IL-6, IL-12(p70), IL-15, and TNF-α) did not differ between the two groups. However, the concentration of IL-18 in the FF of patients in the surgery group was significantly higher than in the non-surgery group (213.92 ± 74.30 pg/mL vs. 145.12 ± 74.20 pg/mL, *P* = 0.01). Thus, surgical removal of endometriotic lesions might stimulate the production of IL-18 in the FF of women with endometriosis.

### Relationship between cytokine levels in follicular fluid and IVF outcomes

Because the levels of IL-18 in FF differed between patients in the surgery and non-surgery groups, we further studied the correlation between IL-18 level and oocyte and embryo quality using Pearson’s bivariate correlation analysis. The levels of IL-18 in the FF samples did not correlate with these cycle parameters (Table 3).

**Table 3:** Pearson’s correlation coefficients between levels of cytokines in FF and IVF outcomes

	No. of mature oocytes/no. of total oocytes (%)		Fertilization rate (%)		No. of high-quality embryos/no. of embryos	
	r	P	r	P	r	P
IL- 18	−0.05	0.75	−0.02	0.89	0.09	0.59

## Discussion

In the present study, we investigated the effect of endometrioma cystectomy on the cytokine profiles in FF and IVF outcomes. The findings demonstrate that surgical treatment prior to IVF had a negative impact on the ovarian reserve and did not improve the clinical pregnancy rate of IVF in women with endometriosis. In addition, our results suggest that surgery for endometriomas might promote increased production of IL-18; however, we failed to find a correlation between IL-18 level and oocyte or embryo quality. Our results add to our understanding of the effect of surgical treatment on the intrafollicular microenvironment in women with endometriosis. The surgery for endometriomas prior to performing artificial reproduction technology (ART) procedures remain controversial. Barri et al. (2010) found that a combined strategy of endoscopic surgery and subsequent IVF led to a significantly higher clinical pregnancy rate than that obtained with IVF alone [26]. Candiani et al. (2018) demonstrated a significant improvement in the AFC of the surgically treated ovary after CO_2_ laser vaporization [27]. However, conflicting results have been reported. Wahd et al. (2014) found that women receiving intracytoplasmic sperm injections following surgery for ovarian endometrioma had poorer clinical outcomes and lower rates of live births compared to women with endometriosis (without previous surgery) and women with no endometriosis [28]. In addition, several studies have found that surgical removal of endometriotic lesions prior to ART treatment does not improve reproductive outcomes, but rather decreases the ovarian reserve [29, 30]. For example, Ata et al. (2017) found that women with endometriomas had a lower ovarian reserve than age-matched controls, and this reserve was further reduced by surgical excision of endometriomas [31]. Several studies have since confirmed that the ovarian reserve damage and the IVF response after surgery was related to the size of the ovarian cysts and the presence of bilateral endometrioma [32–35].

In the current study, we showed that surgical treatment of endometriomas before IVF did not benefit the clinical outcome of IVF and may have decreased the ovarian reserve, serum AMH levels, and AFC. All the surgery-group patients in our study underwent a conventional laparoscopic cystectomy procedure. This type of surgery may decrease the ovarian reserve because it may remove the ovarian cortex and modify the ovarian arterial blood flow [36]. Our results are also consistent with the 2014 ESHRE guidelines recommending clinicians only consider performing cystectomy for endometriomas larger than 3 cm prior to IVF if necessary to improve pelvic pain or to facilitate access to the follicles during oocyte retrieval [37]. Although there was a reduction in the serum AMH level following endometrioma surgery, the pregnancy rates were no different from patients with untreated endometriomas. Porpora et al. (2014) observed that after laparoscopic treatment of ovarian endometriomas, the uterine arterial flow was significantly improved, which seems to have increased the probability of achieving pregnancy [38]. This outcome will be further investigated in our future study.

According to the literature, the presence of endometriomas produces a cytokine imbalance in the peritoneal environment. Additionally, altered production of some cytokines and inflammatory factors in the FF of women with endometriosis has been observed. Monsanto et al. (2016) found that ectopic lesions were major drivers of systemic inflammation in endometriosis and that endometriotic lesion removal significantly altered the inflammatory profile both locally and systemically in women with endometriosis [39]. However, little information is available regarding the alterations of these factors in the FF. In the present study, we found that the levels of chemokines and growth factors were relatively higher than those of inflammatory cytokines in the FF of women with endometriosis-associated infertility. When comparing the surgery group to the non-surgery group, no significant differences were observed in the levels of chemokines, growth factors, or inflammatory cytokines except for IL-18; the levels of IL-18 were significantly higher in patients who underwent surgery prior to IVF treatment.

Our findings are consistent with the relationship between IL-18 and endometriosis identified in other studies. Arici et al. (2003) found measurable levels of IL-18 in the peritoneal fluid of patients receiving GnRH agonists for endometriosis to be significantly higher than those of the control group—patients with endometriosis without treatment [40]. Luo et al. (2006) reported the down-regulation of IL-18 mRNA expression in the ectopic and eutopic endometria of women with endometriosis [41]. In addition, the concentration of IL-18 in the peritoneal fluid was significantly lower in patients with endometriosis than in those without endometriosis [42], suggesting IL-18 might play a pathogenic role in the formation of endometriosis.

Cytokines play an essential but complex role as local regulators of ovarian function, and this role is an area of active investigation. IL-18, initially described as an interferon (IFN) γ inducing factor, plays a central role in the inflammatory cascade and in the process of innate and acquired immunity. It is involved not only in Th1 and NK cell activation, but also in Th2 and Th17 modulation, as well as macrophage activation. IL-18 is known to induce cytokines that are important for both folliculogenesis and ovulation, including IL-1β, TNF-α, and IFN-γ [43]. Salmassi et al. (2017) found that the follicular granulosa cells are the major source of IL-18 and the site of IL-18 receptor expression [43]. The role of IL-18 in oocytes is controversial. Sarapik et al. (2012) demonstrated that IL-18 levels were positively correlated with the number of retrieved oocytes and implantation success in women with different etiologies of infertility [17]. However, conflicting results have been reported. Radwan et al. (2013) did not find any correlation between IL-18 and the number of mature MII oocytes or good-quality embryos in women with “tubal obstruction” subjected to IVF [44]. In agreement with the study of Radwan et al. (2013), we also failed to find a correlation between IL-18 and oocytes or embryo quality in women with endometriosis-associated infertility. Different etiologies of infertility may be one reason for the different outcomes. We therefore hypothesize that surgical intervention might facilitate the expression of IL-18, which may be beneficial to the treatment of endometriosis, but does not improve the quality of oocytes.

Our study has several limitations. Due of the proportion of endometriomas in IVF and the frequently complicated clinical manifestations, it is challenging to collect large numbers of samples from our center. The relativity small sample size is a major limitation of this study, which did not allow us to draw definitive conclusions. Second, ours was a retrospective case-controlled study, and the patients had already undergone surgical excision of their endometriomas before presenting for IVF treatment. As a result, we could not compare their preoperative and postoperative data. Further prospective randomized controlled trials are required.

Taken together, our findings suggest that endometrioma surgery may potentially reduce the ovarian reserve and has little impact on the success rate of IVF. Ovarian endometriomas are not associated with cytokine profiles in FF from infertile women scheduled for IVF, and they are not likely to affect the quality of the oocyte and embryo as a result of an inflammatory mechanism.

## Data Availability

The datasets used and/or analyzed during the current study are available from the corresponding author on reasonable request.

## Abbreviations

AFC: Antral follicle count
AMH: Anti-Müllerian hormone.
ART: Artificial reproduction technology
bFSH: Basal follicle stimulating hormone
BMI: Body mass index
CCL: Chemokine (C-C motif) ligand
CXCL: Chemokine (C-X-C motif) ligand
FF: Follicular fluid
G-CSF: Granulocyte colony–stimulating factor
GM-CSF: Granulocyte-macrophage colony-stimulating factor
Gn: Gonadotrophin
GnRHa: Gonadotropin-releasing hormone agonist
IFN: Interferon
IL: interleukin
IVF: in vitro fertilization
NK: cytotoxic effect of natural killer
TNF: Tumor necrosis factor
VEGF: Vascular endothelial growth factor
